# Patient Use and Experience With Online Access to Electronic Health Records in Norway: Results From an Online Survey

**DOI:** 10.2196/16144

**Published:** 2020-02-07

**Authors:** Paolo Zanaboni, Per Egil Kummervold, Tove Sørensen, Monika Alise Johansen

**Affiliations:** 1 Norwegian Centre for E-health Research University Hospital of North Norway Tromsø Norway; 2 NORCE Norwegian Research Centre Tromsø Norway; 3 Helse Nord IKT Tromsø Norway

**Keywords:** electronic health records, patient online access, patient portals, service utilization, satisfaction, patient empowerment

## Abstract

**Background:**

The electronic health record (EHR) has been fully established in all Norwegian hospitals. Patient-accessible electronic health records (PAEHRs) are available to citizens aged 16 years and older through the national health portal Helsenorge.

**Objective:**

This study aimed at understanding how patients use PAEHRs. Three research questions were addressed in order to explore (1) characteristics of users, (2) patients’ use of the service, and (3) patient experience with the service.

**Methods:**

We conducted an online survey of users who had accessed their EHR online at least once through the national health portal. Patients from two of the four health regions in Norway were invited to participate. Quantitative data were supplemented by qualitative information.

**Results:**

A total of 1037 respondents participated in the survey, most of whom used the PAEHR regularly (305/1037, 29.4%) or when necessary (303/1037, 29.2%). Service utilization was associated with self-reported health, age, gender, education, and health care professional background. Patients found the service useful to look up health information (687/778, 88.3%), keep track of their treatment (684/778, 87.9%), prepare for a hospital appointment (498/778, 64.0%), and share documents with their general practitioner (292/778, 37.5%) or family (194/778, 24.9%). Most users found it easy to access their EHR online (965/1037, 93.1%) and did not encounter technical challenges. The vast majority of respondents (643/755, 85.2%) understood the content, despite over half of them acknowledging some difficulties with medical terms or phrases. The overall satisfaction with the service was very high (700/755, 92.7%). Clinical advantages to the patients included enhanced knowledge of their health condition (565/691, 81.8%), easier control over their health status (685/740, 92.6%), better self-care (571/653, 87.4%), greater empowerment (493/674, 73.1%), easier communication with health care providers (493/618, 79.8%), and increased security (655/730, 89.7%). Patients with complex, long-term or chronic conditions seemed to benefit the most. PAEHRs were described as useful, informative, effective, helpful, easy, practical, and safe.

**Conclusions:**

PAEHRs in Norway are becoming a mature service and are perceived as useful by patients. Future studies should include experimental designs focused on specific populations or chronic conditions that are more likely to achieve clinically meaningful benefits. Continuous evaluation programs should be conducted to assess implementation and changes of wide-scale routine services over time.

## Introduction

### Background

With the rapid rise in the adoption of patient portals, many patients are gaining access to their personal health information online for the first time [1] and expecting extensive access to their health documents [2]. The vast majority of patients endorse the concept of patient-accessible medical records [3]. However, despite the fact that most patients know that they have the legal right to access their records and are interested in what is written, only a minority of them actually access their records [4,5].

An electronic health record (EHR) is the electronic collection of clinical data and can include clinical assessments, laboratory results, radiology findings, nursing documentation, allergy information, medication information, and discharge letters [6]. Patient-accessible electronic health records (PAEHRs) [7] are online services providing patients the ability to view and sometimes edit or comment on their EHR made available by their health care providers [8,9]. Online access to the EHR can be offered to patients, relatives, or other informal carers by health care organizations or on a national scale [6]. PAEHRs can potentially enhance the provision of patient-centered care [10,11], making it easier for most people to understand their health status and health care processes [12]. This may also enable patients to more effectively self-manage and take the lead in consultations [8].

Patients’ increasing demands for medical information, the digitization of health records, and the fast spread of internet access form the basis for introducing new digital health services [13]. At the same time, initiatives to enable patients to access and understand their EHR are gathering momentum [12]. The number and type of documents that are made available online vary between and within countries, making it challenging for patients who visit different health facilities [14]. A recent cross-national comparison reported implementation of PAEHR services in 10 different countries, including Nordic countries, European countries, and non-European countries [15]. In Sweden, access by patients to their EHR was introduced in a pilot county in November 2012 [13]. The PAEHR service has been recently reported to be used nationwide by 19 of the 21 county councils [16], overall with positive experiences for patients [17]. A national patient survey showed that the main reason for use was to gain an overview of one’s health status, and that laboratory results were the most important information to access [18]. The Open Notes pilot study provided patients at three large US health systems access to primary care notes online [4,19]. The great majority of patients reported better understanding of their medical conditions and recall of their treatment plans [20]. In a recent large-scale survey of nearly 23,000 patients who used Open Notes, patients rated note reading as very important for helping take care of their health, feeling in control of their care, and remembering the plan of care [21]. Only a few patients were very confused or more worried after reading notes [21]. The My Health*e*Vet pilot program offered by the US Department of Veterans Affairs was an early prototype allowing patients to view and download content of their EHR, including clinical notes, laboratory tests, and imaging reports [22]. Users were highly satisfied with the service, appreciated the ability to easily access their own EHR, and considered it beneficial to their health and care [23]. In 2012, Australia launched a personally controlled EHR designed around the needs of consumers and aimed at becoming a system-wide activity [24].

### Online Access to Electronic Health Records in Norway

All citizens and residents in Norway have the right to access their health records created by a health care provider (eg, hospital, general practitioner [GP] office, dentist) [25]. The procedure has been that patients could request a copy of their health records on paper or CD from each health care provider for a fee. Upon request, patients are entitled to a brief and simple oral explanation of medical terms. Patients also have the right to know who has accessed or received information from their health records. As a rule, patients have the right to access their entire health record. According to the Patients’ Rights Act, a patient may be denied access to parts of their health record if this is absolutely necessary in order to avoid endangering the patient’s life or causing serious damage to the patient’s health or if access is clearly inadvisable out of consideration for persons close to the patient. A representative of the patient is entitled to obtain the information that the patient is denied access to.

The EHR is fully established by all Norwegian hospitals. The national health portal Helsenorge [26] was established in 2011 to accommodate digital patient services and secure access to health information after secure log-in [27]. In 2012, a white paper, One Citizen–One Record, stated that patients should have online access to their EHR [28]. PAEHR is now offered to citizens aged 16 years and older and to those with parental responsibility for children under the age of 12 years. Online access to the EHR is not yet available for children aged between 12 and 16 years. By October 2016, PAEHR was offered by two (Northern Norway and Western Norway) of the four health regions in Norway through the national portal. Through the service patients can access, read, and download their health records from hospitals (ie, referrals, outpatient visit summaries, clinical notes, discharge letters). Not all documents are available digitally. In Northern Norway, most documents generated after September 2015 are available online, while Western Norway offers online access to documents generated since March 2016. Patients in Northern Norway also can obtain electronic access to older documents upon request. If a citizen has never been to the hospital, no documents appear in the PAEHR. There may also be other reasons why not all of the information is digitally available. Documents can be in a format that is currently not supported (eg, x-rays) or displayed (eg, in the Android app). Some information may not be made available for legal or professional reasons. At the moment, only EHRs from hospitals are available digitally, while health records from GPs, dentists, and other specialists are not. Patients are not notified when new documents are signed and digitally available.

Through the national health portal patients can also retrieve the access log, which shows a list of all those who have accessed their EHR for health or administrative reasons. Use of the PAEHR is not mandatory, and patients can choose not to have their EHR accessible online. The EHR consists of many different types of documents, some of which have been manually scanned. Patients can report errors in the documents to the responsible health care provider so that they can be corrected as soon as possible.

### Study Aim

To date, only a few studies have been performed on large-scale implementation of a national PAEHR and its use by citizens. Evaluations of digital health services are often done from a health care provider perspective, focusing on aspects that are considered important to health care professionals and decision makers. Experiences of evaluations from the perspective of the patients are still scarce [17]. Moreover, most published evaluations have been focused on primary care or office-based practices [29].

This study aimed at understanding how patients use online access to their EHRs through a survey consisting of quantitative data supplemented by qualitative information. In particular, three main research questions were addressed to explore (1) characteristics of the users, (2) patients’ use of the service, and (3) patient experience with the service.

## Methods

### Study Design

We conducted an online survey of users who had activated their personal account at the national health portal and accessed their EHR online at least once. Only citizens with access to the service by October 2016 were invited to participate. These included citizens living in two health regions, Northern Norway and Western Norway. The survey was available after secure log-in on the national health portal. All active users who accessed their EHR online received an invitation through a pop-up window with a brief description of the study and a link to the survey.

The online survey included questions regarding (1) background characteristics, (2) use of the service, and (3) experience with the service (Multimedia Appendix 1). Background characteristics of the users included information on the region in which they were located, gender, age, education level, health care professional background, access to the hospital in the previous year, and self-reported health [30] as defined by the World Health Organization [31]. Use of the service was explored through questions related to frequency of use, number of documents accessible digitally, main reasons for using the service, acquaintance with the service, contact with service support, and availability of older documents. Patient experience with PAEHRs was evaluated with a number of questions concerning ease of access, their opinion about content and features included in the service, its impact on health and treatment, security, overall satisfaction, and future use. Questions on background characteristics and use of the service were multiple choice with a number of alternatives ranging from 2 to 8 depending on the questions. Most of the questions concerning user experiences were scored on a 4-point Likert scale (1=strongly disagree, 2=disagree, 3=agree, 4=strongly agree). Respondents were allowed to skip a question by answering not applicable. Two open-ended questions were included so that respondents could provide additional information regarding their willingness to use the service in the future and whether they would recommend it to others. A third open-ended question was included at the end of the survey to collect additional comments provided by the users.

The online survey was developed by the Norwegian Centre for E-health Research in collaboration with the project implementing the PAEHR service in Northern Norway on behalf of the Northern Norway Regional Health Authority. The survey was published on the national portal by the Norwegian Directorate of eHealth. The link to the survey was available for a period of 4 weeks. All information collected through the survey was anonymous and not personally identifiable. Participation in the survey was based on consent wherein each respondent could choose not to answer the questionnaire. Ethics approval from the Regional Committees for Medical and Health Research Ethics was deemed not necessary according to the Health Research Act on medical and health research entered into force in Norway in 2009. The study was approved by the Data Protection Officer of the University Hospital of North Norway. The Checklist for Reporting Results of Internet E-Surveys (CHERRIES) was used to develop the survey and report its results [32]. The online survey was developed with the online data collection solution Questback Essentials, and its technical functionality was tested before being published.

### Data Analysis

Respondents were analyzed by age according to the following groups: 16 to 24 years, 25 to 34 years, 35 to 44 years, 45 to 54 years, 55 to 64 years, and over 65 years. Population data for the year 2015 were provided by the Center for Clinical Documentation and Evaluation and used to compare the demographic characteristics of the respondents with the general population and patients receiving specialist health care. Participation and completion rates were not reported, as data on unique visitors were not available. The selection of respondents to this survey was assumed to be representative of those who actually used the service.

Data on patient use and experience with the service were summarized by descriptive statistics as well as by graphs. In the analysis of the questions concerning user satisfaction with the service, results were summarized by the proportion of respondents who agreed with a certain aspect (scores 3 and 4) and those who disagreed (scores 1 and 2). Possible variations in service utilization among respondents were explored by analyzing frequency of use (light users vs regular users) against patient characteristics. A Pearson Chi-square test was used to explore associations between the two categorical variables.

Qualitative data provided in the open text fields were subject to content analysis [33]. These open text fields were not mandatory. The information was provided only by those respondents who were willing to express additional comments about the service. These could include general statements, positive feedback, criticism, reports of technical problems, and suggestions for service improvements. Answers were stratified into positive, neutral, and negative. The content of these answers was analyzed by a multidisciplinary research team consisting of two authors. Codes were assigned to each comment. The coding labels were compared to find similarities in the interpretations of the content and resolve differences. The results were summarized around common themes. Qualitative data were used to support the results of the quantitative data. Comments providing good examples of patient opinions around the different themes are presented.

Data analysis was performed by NORCE Northern Research Centre and the Norwegian Centre for E-health Research. Data were extracted in Excel (Microsoft Corp) and further analyzed in SPSS Statistics version 25 (IBM Corp) and R version 3.4.2 (R Foundation for Statistical Computing).

## Results

### Characteristics of the Users

The online survey was available on the national portal from October 24, 2016, to November 21, 2016. In total, 1037 users answered the survey. Of these, 569 respondents (54.9%) were from Western Norway, 395 respondents (38.1%) were from Northern Norway, and 73 respondents (7.0%) had received health care in both regions (Table 1).

**Figure 1:**
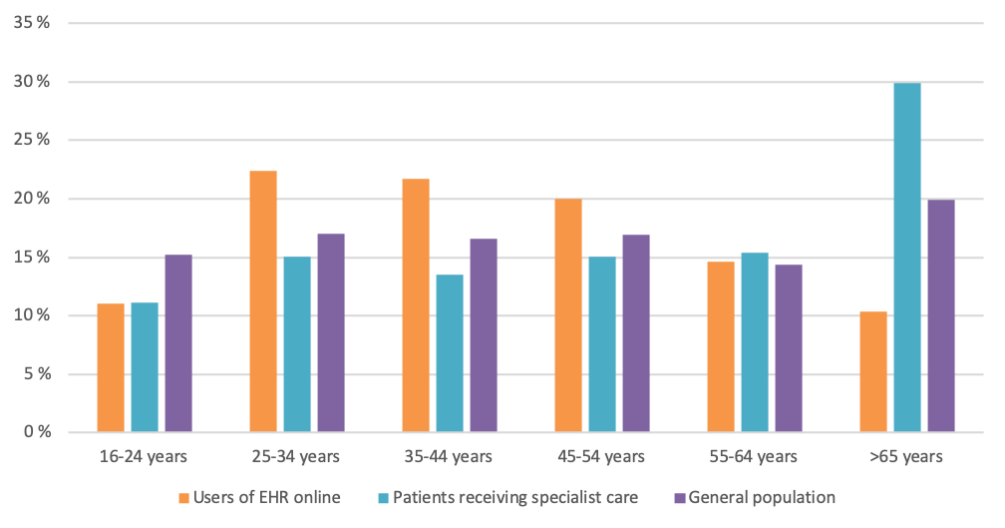
Distribution of users by age groups compared with patients receiving specialist health care and the general population.

Respondents were almost equally distributed by gender, with a slightly higher proportion of female users. Users in all age groups accessed their EHR online. Use of the service was higher for people aged 25 to 54 years (ie, citizens in their prime working lives). Access was lower for citizens in the age group over 65 years compared with the general population and those receiving specialist health care (Figure 1).

Only 9.3% (96/1037) of the respondents had an education at primary or secondary school level. Almost half of the users (491/1037, 47.3%) had an education at university level or higher. About a third of the respondents had a health care professional background.

About half of the respondents described their health status as good, while 18.6% (193/1037) considered themselves to be in poor health. Overall, 90.3% (937/1037) of the users reported to have sought a doctor (including hospitalizations) at least once in the previous year.

**Table 1:** Characteristics of the users.

Characteristic			Value, n (%)
**Region (n=1037)**			
	Northern Norway	395 (38.1)	
	Western Norway	569 (54.9)	
	Both regions	73 (7.0)	
**Gender (n=1037)**			
	Male	447 (43.1)	
	Female	590 (56.9)	
**Age in years (n=1037)**			
	16-24	114 (11.0)	
	25-34	232 (22.4)	
	35-44	225 (21.7)	
	45-54	207 (20.0)	
	55-64	152 (14.6)	
	Over 65	107 (10.3)	
**Education (n=1037)**			
	Primary school	11 (1.1)	
	Secondary school	85 (8.2)	
	Technical school	55 (5.3)	
	High school	395 (38.1)	
	University	475 (45.8)	
	Doctoral degree	16 (1.5)	
**Health care professional background (n=1037)**			
	Yes	266 (25.7)	
	No	771 (74.3)	
**Self-reported health (n=1037)**			
	Very good	165 (15.9)	
	Good	361 (34.8)	
	Moderate	283 (27.3)	
	Bad	159 (15.3)	
	Very bad	34 (3.3)	
	N/A	35 (3.4)	
**Sought a doctor in the past year (n=1037)**			
	Yes	937 (90.3)	
	No	64 (6.2)	
	N/A	36 (3.5)	
**Number of doctor’s visits in the past year (n=702)**			
	1-5	365 (52.0)	
	6-10	200 (28.5)	
	11-20	62 (8.8)	
	Over 20	75 (10.7)	

### Patient Use of the Service

About a third of the respondents (305/1037, 29.4%) accessed their EHR online regularly, and a similar proportion of respondents (303/1037, 29.2%) used the service when necessary (Table 2). The remaining users accessed the service only once or twice. The majority of the users (601/1037, 58.0%) had up to 50 documents available online, while fewer users (177/1037, 17.0%) had more than 50 documents. Only a fourth of the respondents tried the service without having any documents online. About two-thirds (516/778, 66.4%) of those who had documents available had accessed at least 80% of them.

The vast majority of users accessed their EHRs online to look up health information received from the health care provider (687/778, 88.3%) or to keep track of their treatment (684/778, 87.9%). Another important reason for using the service was to prepare for an appointment or a hospital admission. Patients also considered it useful to share documents with their GP, other health care professionals, family, or friends.

Over half of the respondents (432/778, 55.5%) found the service while exploring another section of the national portal [26]. The remaining users became acquainted with the service from other sources, including media, health care professionals, or information provided at the hospital. Contact with service support occurred for 15.3% (119/778) of the users. Reasons included request to access older documents, report of incorrect or missing information, or need for explanation. Of those who requested older documents, 35.9% (14/39) obtained access after contacting service support.

The analysis of service utilization against patient characteristics (Table 3) revealed that frequency of access to PAEHR was associated with health region (*P*<.001), age (*P*=.02), gender (*P*<.001), health care professional background (*P*=.004), self-reported health (*P*<.001), and attendance to a doctor in the previous year (*P*<.001). In particular, post hoc tests showed that the proportion of regular users was higher among patients living in Northern Norway, women, those with a health care professional background, patients in moderate to very bad health status, and those who had doctor’s visits in the past year. Conversely, the number of light users was higher among patients living in Western Norway, men, citizens aged 16 to 25 years, patients in very good health status, and those who did not seek the doctor during the previous year. Frequency of use was also found to be associated with the number of documents available online (*P*<.001), with post hoc test showing that the number of light users was higher among those who did not have any documents available online.

**Table 2:** Patient use of online access to electronic health records.

Patient use of the service		Value, n (%)
**Frequency of use (n=1037)**		
	First time	283 (27.3)
	A couple of times	146 (14.1)
	When needed	303 (29.2)
	Regularly	305 (29.4)
**Number of documents available online (n=1037)**		
	None	259 (25.0)
	1-50	601 (58.0)
	50-99	96 (9.2)
	100-499	60 (5.8)
	>500	21 (2.0)
**Documents opened (n=778)**		
	Less than 15%	88 (11.3)
	15%-49%	78 (10.0)
	50%-79%	96 (12.3)
	80%-99%	206 (26.5)
	100%	310 (39.9)
**Main reasons for using the service (n=778)**		
	Look up health information	687 (88.3)
	Keep track of the treatment	684 (87.9)
	Prepare for an appointment or admission	498 (64.0)
	Share documents with GP^a^ or other health care professionals	292 (37.5)
	Share documents with family and friends	194 (24.9)
**Acquaintance with the service (n=778)**		
	Helsenorge	432 (55.5)
	Media (newspaper, radio, TV, social media, etc)	129 (16.6)
	Health care professionals	115 (14.8)
	Written information at the hospital	110 (14.1)
	Other	76 (9.8)
	Family or friends	72 (9.3)
**Contact with service support (n=778)**		
	Yes	119 (15.3)
	No	659 (84.7)
**Availability of older documents (n=39)**		
	Yes	14 (35.9)
	No	25 (64.1)

^a^GP: general practitioner.

**Table 3:** Association between service utilization and patient characteristics.

Patient characteristics		Light user^a^, n (%)	Regular user^b^, n (%)	*P* value
**Region**				**<.001**
	Northern Norway (n=395)	129 (32.7)	266 (67.3)	
	Western Norway (n=569)	251 (44.1)	318 (55.9)	
**Gender**				**<.001**
	Male (n=447)	215 (48.1)	232 (51.9)	
	Female (n=590)	214 (36.3)	376 (63.7)	
**Age in years**				**.02**
	16-24 (n=114)	63 (55.3)	51 (44.7)	
	25-34 (n=232)	102 (44.0)	130 (56.0)	
	35-44 (n=225)	87 (38.7)	138 (61.3)	
	45-54 (n=207)	79 (38.2)	128 (61.8)	
	55-64 (n=152)	54 (35.5)	98 (64.5)	
	Over 65 (n=107)	44 (41.1)	63 (58.9)	
**Education**				**.48**
	Primary school (n=11)	5 (45.5)	6 (54.5)	
	Secondary school (n=85)	29 (34.1)	56 (65.9)	
	Technical school (n=55)	22 (40.0)	33 (60.0)	
	High school (n=395)	158 (40.0)	237 (60.0)	
	University (n=475)	210 (44.2)	265 (55.8)	
	Doctoral degree (n=16)	5 (31.3)	11 (68.8)	
**Health care professional background**				**.004**
	Yes (n=266)	90 (33.8)	176 (66.2)	
	No (n=771)	339 (44.0)	432 (56.0)	
**Self-reported health**				**<.001**
	Very good (n=165)	109 (66.1)	56 (33.9)	
	Good (n=361)	157 (43.5)	204 (56.5)	
	Moderate (n=283)	90 (31.8)	193 (68.2)	
	Bad (n=159)	52 (32.7)	107 (67.3)	
	Very bad (n=34)	9 (26.5)	25 (73.5)	
**Sought a doctor (past year)**				**<.001**
	Yes (n=937)	354 (38.0)	583 (62)	
	No (n=64)	54 (84)	10 (16)	
**Number of documents available online**				**<.001**
	None (n=259)	223 (86.1)	36 (13.9)	
	1-50 (n=601)	189 (31.4)	412 (68.6)	
	50-99 (n=96)	8 (8.3)	88 (91.7)	
	100-499 (n=60)	6 (10.0)	54 (90.0)	
	>500 (n=21)	3 (14.3)	18 (85.7)	

^a^Used the service for the first time/a couple of times.

^b^Used the service when needed/regularly.

### Patient Experience With the Service

The vast majority (965/1037, 93.1%) of the users found it easy to access their EHR online (Table 4). Of those users who had difficulties in accessing the service, only 15.3% (11/72) sought help from family, friends, service support, or health personnel.

**Figure 2:**
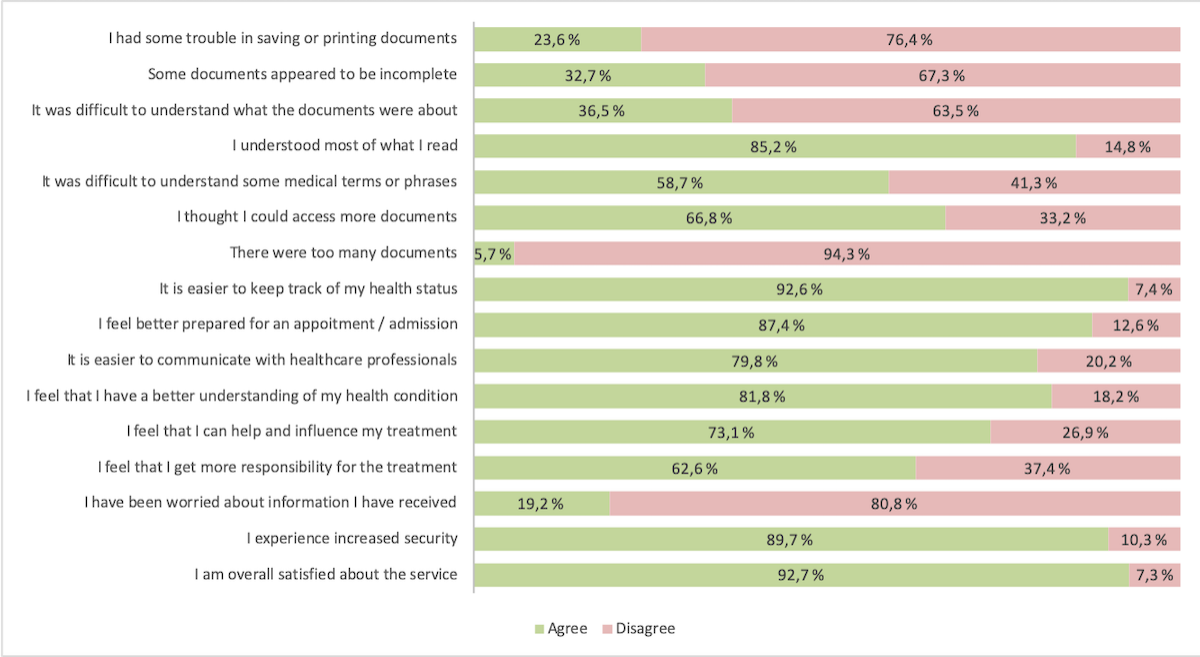
Patient satisfaction with online access to electronic health record.

About two-thirds of respondents (476/713, 66.8%) expected to have more documents accessible through the service, while only a small percentage of patients (40/703, 5.7%) thought that there were too many documents (Figure 2). There were some difficulties in understanding what all the documents listed in their EHR were about. However, the vast majority of the users (643/755, 85.2%) understood most of the content reported in the documents, despite over half of them (430/733, 58.7%) acknowledging difficulties in understanding some medical terms or phrases. There were also a number of respondents (199/608, 32.7%) who thought that some documents were incomplete. Only a fourth of the users (99/419, 23.6%) encountered technical challenges in saving or printing documents that were available digitally.

Clinical advantages to the patients included a better understanding of their health condition (565/691, 81.8%) and easier control of their health status (685/740, 92.6%). After using the service, most users acknowledged that they felt better prepared for future hospital visits or admissions (571/653, 87.4%) and that it became easier to communicate with health care professionals at the hospital (493/618, 79.8%). Patients also experienced increased empowerment. They felt more responsible for their treatment (413/660, 62.6%) and thought that they could better influence its progress (493/674, 73.1%). Only a small proportion of patients (136/707, 19.2%) expressed concerns about the information accessible online. Users also experienced better security (655/730, 89.7%) when accessing their EHR online.

The overall satisfaction with the service was very high (700/755, 92.7%). The vast majority of the respondents stated that they would continue accessing their EHR online in the future (753/778, 96.8%) and they recommended the service to others (695/778, 89.3%; Table 4).

**Table 4:** Accessibility and patient preferences with online access to electronic health record.

Patient experience with the service		Value, n (%)
**Ease of access (n=1037)**		
	Very easy	559 (53.9)
	Easy	406 (39.2)
	Difficult	52 (5.0)
	Very difficult	20 (1.9)
**Sought for help (n=72)**		
	Yes	11 (15.3)
	No	61 (84.7)
**Future use of the service (n=778)**		
	Yes	753 (96.8)
	Maybe	18 (2.3)
	No	7 (0.9)
**Recommend the service to others (n=778)**		
	Yes	695 (89.3)
	Maybe	72 (9.3)
	No	11 (1.4)

### Qualitative Feedback on the Service

A total of 268 comments, most of which were positive (252/268, 94.0%), were provided in the open text field following the question related to willingness to use the PAEHR in the future. The main reason (203/268, 75.7%) why respondents would continue accessing their EHR online was related to the perceived impact of the service. Patients reported that the PAEHR helped them to gain a better understanding of their health status, obtain a more comprehensive overview of hospital access, and follow their treatment more closely. This was particularly important for patients with complex, long-term, or chronic conditions.

This [service] has a great value to me as a patient. Now I have a much better picture of my own disease than before. I often have visits with specialists who are not very communicative, and now I have the opportunity to prepare questions—and the best expert on my own illness is myself. Why didn't this service come before?

Patients also appreciated the chance to easily read all the information that health personnel wrote about them after attending visits, thus becoming more confident in understanding it, reporting mistakes or misunderstandings, and being better prepared for future visits.

I am under psychiatric evaluation. By accessing the health records between visits I can see if the health personnel has misunderstood something I have said. This can be clarified during the next consultation. When the health personnel writes things which have not been discussed yet, I can be better prepared for the next consultation. The service therefore makes the treatment more effective and more appropriate.

There were also 23 comments (8.6%) regarding practical benefits of using the service. Patients especially appreciated the convenience of accessing their EHRs directly from home, where they could easily find all their digital documents in one place and read them in a peaceful environment. The remaining comments were related to positive feedback of a more general nature (21/268, 7.8%), criticism (16/268, 6.0%), or additional information on health status (5/268, 1.9%).

In the second open text field following the question on whether respondents would recommend using the service to others, a total of 208 comments were expressed, most of which were positive opinions (197/208, 94.7%). Online access to EHRs were described as useful, informative, effective, helpful, easy, practical, and safe.

I think that this service is especially good when you have old parents or very sick family members who do not get all the information when they are at the doctor or at the hospital. A relative can then get permission to read and try to understand the content and follow up with the treatment (for instance, hospitals admissions, etc.). Everything is all gathered here, instead of having papers around your house.

Another advantage perceived by the users was that the PAEHR increased accessibility compared with the traditional practice of requesting a copy of their health records on paper or CD. This, in turn, contributed to improved patient engagement.

Many are interested in what is written in their health record but just not enough to make them ask to get access to it. Through online access it becomes easier for most people to keep themselves up to date on their own health record, as well as on future appointments.

There were 2.4% of respondents (5/208) who expressed mixed comments regarding the utility of the service, which could be more or less beneficial depending on the user characteristics (eg, age, computer literacy) as well as their health condition. There were only 2.9% of comments (6/208) expressing concerns about online access to EHR, some of which was pointed out by users with a health care professional background.

Online access to the health record should not be open to everyone. Now I think first of all about psychiatric patients. I think it can be negative and cause distrust toward health personnel, making them feel like patients and not like persons (due to the way things are formulated and professional expressions). Several of the patients I talk with feel unheard and trust much less in the treatment and health care providers than before...Health professionals also express uncertainty and dissatisfaction with open access to health records.

Finally, 129 comments were provided in the open-ended question included at the end of the survey where users could write additional thoughts. Four common themes were identified after analyzing the content of these answers: availability of documents, information about their health status, technical issues and suggestions for improvement, and experienced satisfaction. There were 36.4% of comments (47/129) concerning the availability of documents online. Some users missed the chance to access older documents, health records from their GP or other health professionals, documents for their children, laboratory test results, and digital imaging tests. There was also a number of comments about the current lack of documents from the two other health regions which had not yet implemented online access to EHRs. Other respondents reported that they had no or little information visible in their PAEHR. A total of 36.4% of users (47/129) voluntarily provided comments with general information about their health status. There were 13 users who underwent cancer treatment, and 16 users who referred to the presence of chronic illness, such as rheumatologic diseases and other musculoskeletal conditions. Other comments were related to different long-lasting conditions, health problems under treatment, or simply additional information about the number of visits to the hospital. There were 17.8% (23/129) comments specifically reporting issues of a technical nature encountered while using the service. Most comments were related to difficulties in opening specific types of documents and file formats, using a mobile phone, logging in, or accessing specific features. Features which could be improved were the possibility of retrieving the access log, marking documents read and unread, and asking to modify or delete documents. Some respondents also suggested new functions. There were, for instance, four users who expressed their wish for a feature where they could register themselves as blood, organ, or body donors. Finally, 9.3% of comments (12/129) included feedback regarding general satisfaction with the service and its benefits for patients, such as a better understanding of their own health condition. Two users expressed some concerns related to how the communication with health personnel changed after accessing their EHR online.

## Discussion

### Principal Findings

The results obtained from this survey showed that PAEHR in Norway is becoming a mature and useful service. Most of the users accessed their EHR online regularly, for instance when new information became available after a hospital appointment, and read most of the digital documents. The vast majority of the users had at least one doctor's visit in the previous year, meaning that they had digital documents which were recently made available online. There were fewer patients who tried the service for the first time, some of whom did not have any documents accessible. Service utilization for users in Northern Norway was higher than for those in Western Norway, reflecting the earlier implementation of the service in that region.

The findings of this study seemed to be aligned with the most recent version of Andersen's behavioral model used to analyze utilization of health care services based on contextual as well as individual determinants of access to medical care [34]. In particular, the following components were found to affect utilization of the PAEHR: (1) predisposing factors, including demographic characteristics (eg, age, sex), social factors (eg, education), and mental factors (eg, attitudes), and (2) need factors, comprising both perceived need for health services (ie, how people experience their own health) and evaluated need (ie, professional assessments). Enabling factors, including financing (eg, income) and organizational factors (eg, transportation) were not covered by this survey, and therefore no association with service utilization could be explored. In this survey, frequency of access to the PAEHR was found to be associated with self-reported health status, region, gender, age, and health care professional background. The service appeared to be more suitable to patients in need of medical care, especially those in moderate or bad health and greater overall morbidity, as suggested by other studies [11,35]. Patients with multiple chronic conditions have, in fact, significantly higher odds of accessing their records [36]. Despite users in all age groups accessed their EHR online, citizens in the age group over 65 years used the service at a lower degree compared with patients receiving specialist health care and the general population. One explanation is that older patients tend to have a lower computer literacy and thus are less likely to use digital services [37], especially when accessing them for the first time [1]. Another explanation is that older patients are often sicker, with a higher risk of having health conditions that can affect their ability to use technology and interpret digital content [36,38]. However, it is suggested that those who can benefit the most from a PAEHR may be the least able to use it [39]. It is therefore important to address this patient group so that more elderly will be able to access their EHRs in the future. About 60% of those users over 65 years used the service regularly. Most first-time users were found in the age group 16 to 25 years. In our survey, adult females were the most active users of PAEHRs. Similar findings were found in recent large-scale studies [18,21]. One reason might be the general lower consultation rate among men [40]. Users with a health care professional background used the service at a higher degree, confirming the results from the use of PAEHRs in Sweden [18]. In a European study on citizens' use of eHealth services across seven countries, women and people with higher education tended to use the internet more for health purposes [41].

Most respondents indicated that the system was easy to use, confirming the positive findings from other studies on patient experience with PAEHRs [8,29,42,43]. Two-thirds of the respondents expected to have more documents accessible online. As previously mentioned, not all of the information was digitally available. Health records from GPs, dentists, and other specialists were not yet accessible through the service. Some documents were not made available for technical, legal, or professional reasons. Moreover, only documents generated after the PAEHR was introduced in Northern Norway and Western Norway were available online. Some users also complained about the lack of documents from the two other regions which had not implemented the service. Approximately one-third of all respondents thought that some documents were incomplete. Similar results have been previously reported [35,43]. Despite some difficulties in understanding specific medical terms or phrases, the vast majority of the users understood most of the content reported in their EHR, confirming findings from other studies [5,42]. Technical challenges and issues related to security and confidentiality reported in previous studies [29,43,44] affected only a minority of users and did not seem to represent a barrier affecting service utilization. However, some users pointed out a number of technical issues that could be improved and suggested new features that could be added to the service.

Patients using PAEHRs in Norway perceived a number of clinical benefits that were also found in other studies, including enhanced knowledge of their health and improved self-care [11,21,22,35,42,45], greater patient empowerment [9,21,22], and easier communication with health care providers [11,22,35,42,45]. The vast majority of the users also experienced increased security [11]. There were, however, a few users who expressed concerns about use of the service by elderly with low computer literacy as well as by patients with severe health conditions, who might prefer accessing new information only after having communicated directly with health personnel. The results obtained from the analysis of the qualitative data confirmed that the PAEHR was particularly useful to patients with complex, long-term, or chronic conditions. Despite some health professionals expecting access to health records to be harmful, patients who choose to look at their documents often find access helpful and reassuring even if the news is bad, such as in cases of cancer care [46]. Through online access it becomes easier for most people to look up health information received from the health care provider [45], take care of their treatment [21], prepare for an appointment or a hospital admission [45], and share documents with someone else [21]. Few users with a health care professional background thought that online access to the health records should not be open to everyone. One respondent, for instance, expressed worries for psychiatric patients, who could feel unheard and trust much less in their health care providers than before. Overall, over 90% of the users indicated that they would continue using the service in the future and recommend it to others, confirming findings from other studies [18,23,27].

### Strengths and Limitations

With a total of 1037 respondents, this survey is one of the few large-scale studies focusing on patient experience with PAEHRs. We were able to collect a large amount of quantitative data from multiple choice questions and use them to describe the characteristics of the users, patient use of the service, and patient experience with the service. Moreover, quantitative data were used to explore the association between different variables and especially how patient characteristics affected service utilization. However, this was mainly a descriptive survey rather than an explorative study. For a robust investigation of the factors affecting service utilization, a more comprehensive data collection process would be needed. Qualitative information was also collected from three open text fields. A total of 605 comments were analyzed and used to support the quantitative data. Users providing additional comments tend to be those who have very positive or negative experiences. To collect more detailed information on relevant topics, such as patient empowerment, in-depth qualitative interviews with randomly selected users should be conducted in future studies.

Despite the number of respondents, one main limitation of this study is related to its design. Although observational studies and surveys have provided evidence of benefits and satisfaction for patients, there is still little reliable evidence of improved health outcomes from experimental studies [37]. Future evaluations of PAEHRs should focus on specific populations or chronic conditions that are more likely to achieve clinically meaningful benefits and use randomized controlled trials or implementation research methods [37,47].

This was one of the largest surveys conducted on the use of PAEHRs, with respondents from two of the four health regions in Norway. By 2019, online access to EHRs will be offered to citizens in South-Eastern Norway, meaning that an even larger proportion of the population will have access to the service. Patient experience with the service might be influenced by a different level of maturity of the service and therefore vary across regions. For such a wide-scale routine service, whose functionalities might change over time, it is important to implement continuous evaluation programs able to simultaneously evaluate digital health interventions while they are being designed, developed, and deployed [48]. Finally, this survey was limited to patients who accessed the service at least once. Moreover, 25% of the respondents did not have any documents available online. Future studies might be focused on exploring the reasons why some patients do not use the service.

### Conclusions

We conducted an online survey of users of the PAEHR in Norway. A total of 1037 respondents participated in the survey, most of whom accessed their EHRs online regularly. Service utilization was associated with self-reported health, age, gender, education, and health care professional background. Patients were highly satisfied with the service and found it useful to look up health information, keep track of their treatment, prepare for a hospital appointment, and share documents with their GP or family. Users also experienced clinical benefits from accessing their EHR online, including enhanced knowledge of their health, improved self-care, greater empowerment, easier communication with health care providers, and increased security. Future studies should include both experimental designs focused on specific populations or chronic conditions that are more likely to achieve clinically meaningful benefits and continuous evaluation programs to evaluate implementation and changes of wide-scale routine services over time.

## Appendix

Multimedia Appendix 1 Online survey (in Norwegian).

## Abbreviations

CHERRIES: Checklist for Reporting Results of Internet E-Surveys
EHR: electronic health record
GP: general practitioner
PAEHR: patient-accessible electronic health record
