# Supplementary material for: Patient Use and Experience With Online Access to Electronic Health Records in Norway: Results From an Online Survey
Source: J Med Internet Res. 2020 Feb 7;22(2):e16144. doi: 10.2196/16144 (PMC7055829; doi:10.2196/16144)
Supplement: Multimedia Appendix 1 [file jmir_v22i2e16144_app1.pdf]

Spørreskjema til brukere av «Pasientjournal» på helsenorge.no

Til deg som har brukt tjeneste «Pasientjournal» på den nasjonale helseportalen helsenorge.no.

Dette er en ny tjeneste i Norge, og vi ønsker derfor å stille deg noen spørsmål slik at vi kan forbedre tjenesten. Vi ønsker å vite *hva du synes om tjenesten og hvordan du bruker den.* Vi trenger også litt bakgrunnsinformasjon om deg. Resultatene fra undersøkelsen vil brukes i rapporter og forskningsartikler.

Undersøkelsen er anonym, og det vil ikke være mulig å identifisere deg. Det tar omtrent 10–12 minutter å svare.

1) * Hvordan vil du betegne din bruk av tjenesten «pasientjournal»?

- ○ Jeg bruker den regelmessig
- ○ Jeg bruker den ved behov
- ○ Jeg har vært inne et par ganger for å se
- ○ Dette var første gang

2) * Hvordan var det å finne fram til tjenesten «pasientjournal» etter innlogging på helsenorge.no?

- ○ Veldig enkelt
- ○ Ganske enkelt
- ○ Vanskelig
- ○ Veldig vanskelig

3) * Har du tatt kontakt med noen for å få hjelp til å finne fram?

- ☐ Nei
- ☐ Ja, med 800Helse
- ☐ Ja, med prosjektet
- ☐ Ja, med helsepersonell
- ☐ Ja, med venner og kjente

4) * Omtrent hvor mange dokumenter ser du i «pasientjournal» på helsenorge.no?

- ○ Flere enn 1000
- ○ Mellom 500–999
- ○ Mellom 100–499
- ○ Mellom 50–99
- ○ Under 50
- ○ Ingen som jeg kunne se elektronisk

5) * Omtrent hvor stor andel av dokumentene har du åpnet («klikket på»)?

- ○ Alle (100 %) av dokumentene i lista
- ○ De fleste (80 %–99 %)
- ○ Over halvparten (50 %–79 %)
- ○ Under halvparten (15 %–49 %)
- ○ Noen få (under 15 %)

6) * Har du kontaktet behandler eller behandlingssted med spørsmål om din eller barns journal?

- ☐ Ja, jeg har bedt om innsyn i eldre dokumenter
- ☐ Ja, jeg har meldt feil eller mangler i journalen
- ☐ Ja, jeg har bedt om forklaring på ting jeg ikke forstod
- ☐ Nei, det har ikke vært aktuelt
- ☐ Nei, det var vanskelig å finne ut av hvem jeg skulle kontakte

7) * Har du fått digital tilgang til eldre dokumenter?

- ○ Ja
- ○ Delvis
- ○ Nei

8) * Hvor enig er du i følgende påstander

	Helt uenig	Delvis uenig	Delvis enig	Helt enig	Ikke aktuell
Jeg har hatt problemer med å lagre eller skrive ut dokumentene	○	○	○	○	○
Noen dokumenter så ut til å være ufullstendige	○	○	○	○	○
Jeg synes det var vanskelig å forstå hva de ulike dokumentene handlet om uten å åpne selve dokumentet	○	○	○	○	○
Jeg forstod det meste av det jeg leste	○	○	○	○	○
Jeg synes det var vanskelig å forstå noen medisinske ord eller uttrykk	○	○	○	○	○
Jeg trodde jeg skulle få tilgang til flere dokumenter enn det jeg fikk	○	○	○	○	○
Jeg synes det var for mange dokumenter	○	○	○	○	○
Jeg opplever at det er enklere å holde oversikt over min helsetilstand når jeg har tilgang til journalen	○	○	○	○	○
Jeg mener at jeg er bedre forberedt til time / innleggelse etter at jeg tok i bruk «pasientjournal»	○	○	○	○	○
Jeg synes det er lettere å kommunisere med helsepersonell etter at jeg tok i bruk «pasientjournal»	○	○	○	○	○
Etter at jeg tok i bruk «pasientjournal», opplever jeg at jeg har en bedre forståelse av min helsetilstand	○	○	○	○	○
Jeg opplever at jeg kan være med og påvirke min behandling	○	○	○	○	○
Jeg opplever at jeg får mer ansvar for behandlingen	○	○	○	○	○
Jeg har blitt urolig over informasjon jeg har fått gjennom «pasientjournal»	○	○	○	○	○
Jeg opplever økt trygghet gjennom «pasientjournal»	○	○	○	○	○
Alt i alt synes jeg tjenesten fungerte tilfredsstillende	○	○	○	○	○

9) * Har du gjort noe av det følgende?

	Sjelden eller aldri	Noen ganger	Ofte
Jeg bruker «pasientjournal» til å forberede meg til en time eller innleggelse	○	○	○
Jeg bruker «pasientjournal» til å slå opp informasjon jeg har fått fra behandler	○	○	○
Jeg bruker «pasientjournal» til å følge med på behandlingen	○	○	○
Jeg deler (viser) dokumenter fra «pasientjournal» med familie og venner	○	○	○
Jeg deler journaldokumenter med fastlegen min eller annet helsepersonell	○	○	○

10) * Jeg ble oppmerksom på tjenesten «pasientjournal» gjennom

- ☐ Min behandler eller annet helsepersonell
- ☐ Skriftlig informasjon på sykehuset/behandlingsstedet
- ☐ Familie eller venner
- ☐ helsenorge.no
- ☐ Medier (aviser, radio, TV, Facebook, etc.)
- ☐ Annet ________

11) * Tror du at du vil bruke «pasientjournal» seinere?

- ○ Ja
- ○ Nei
- ○ Vet ikke

12) Begrunnelse:

13) * Vil du anbefale tjenesten «pasientjournal» til andre?

- Ja
- Nei
- Vet ikke

14) Begunnelse:

15) * Hvilken helseregion har du vært i kontakt med?

- Helse Nord
- Helse Vest
- Helse Nord og Helse Vest

16) * Kjønn:

- Kvinne
- Mann

17) * Alder:

- Under 24 år
- 25 - 34 år
- 35 - 44 år
- 45 - 54 år
- 55 - 64 år
- Over 65 år

18) * Høyeste fullførte utdanning:

- Barneskole (6. eller 7. skoleår) / Folkeskolenivå
- Ungdomsskole (9. eller 10. skoleår) / Framhaldskole / Realskolenivå
- Videregående opplæring, yrkesfag
- Videregående opplæring, allmenfag
- Teknisk fagskole, arbeidsmarkedsopplæring
- Høyskole / Universitet 1-3 år (Bachelor, cand.mag, høgskolekandidat, sykepleier, lærer, ingeniør, osv)
- Høgskole / Universitet 4-5 år (Master, hovedfag, sivilingeniør, osv)
- Doktorgrad

19) * Har du helsefaglig utdanning og/eller arbeider du i helsesektoren?

- Ja
- Nei

20) * Har du oppsøkt lege i løpet av de siste 12 månedene? (dette inkluderer sykehusinnleggelser og legevakt, men IKKE tannlegetimer)

- Ja, jeg har selv oppsøkt lege
- Ja, jeg har oppsøkt lege på vegne av andre
- Ingen ganger
- Vet ikke / ønsker ikke å svare

21) Hvis ja, hvor mange ganger?

22) * Hvordan er helsen din stort sett?

- ○ Svært god
- ○ God
- ○ Middels
- ○ Dårlig
- ○ Svært dårlig
- ○ Vet ikke / ønsker ikke å svare

23) Andre kommentarer:

© Copyright www.questback.com. All Rights Reserved.
